# Cost-Effectiveness of App-Guided Self-Management for Posttraumatic Stress: Trial-Based Economic Evaluation

**DOI:** 10.2196/69426

**Published:** 2025-09-18

**Authors:** Jonathan Siverskog, Johannes Andersson, Ida Hensler, Erik Grönqvist, Filip K Arnberg

**Affiliations:** 1 Centre for Health Economic Research (HEFUU) Uppsala University Uppsala Sweden; 2 Economics Department of Management and Engineering Linköping University Linköping Sweden; 3 Department of Economics Uppsala University Uppsala Sweden; 4 National Centre for Disaster Psychiatry Department of Medical Sciences Uppsala University Uppsala Sweden; 5 Department of Clinical Neuroscience Karolinska Institutet Stockholm Sweden; 6 Health Economics Department of Medical Sciences Uppsala University Uppsala Sweden

**Keywords:** economic evaluation, cost-effectiveness, posttraumatic stress, posttraumatic stress disorder, PTSD, app, self-management

## Abstract

**Background:**

App interventions show promise as effective interventions for trauma-related distress, but evaluations of their cost-effectiveness are scarce.

**Objective:**

This study aimed to assess the cost-effectiveness of an app-based intervention for self-management of posttraumatic stress compared to no guided self-management.

**Methods:**

An economic evaluation from a Swedish public health care perspective was conducted alongside a randomized controlled trial in which participants (N=179) were randomly assigned to either immediate exposure (intervention group; n=89, 49.7%) or delayed exposure at 3 months (waitlist group; n=90, 50.3%). The number of quality-adjusted life years (QALYs) gained or lost, increases or decreases in the use of different types of health care, and the monetary costs (in SEK; 2023 price level) saved or incurred with the intervention versus the comparator at 9 months after exposure were estimated based on functional disability and health care consumption reported by participants via a web-based written questionnaire at baseline and at 3, 6, and 9 months of follow-up. Estimation was done via linear regression with clustering at the participant level. The probability of the intervention being cost-effective was calculated over a range of cost-effectiveness thresholds up to SEK 1 million per QALY (US $94,225 per QALY; 2023 average exchange rate, US $1=SEK 10.61), and value of information analysis was used to interpret statistical uncertainty in the cost-effectiveness results.

**Results:**

There was no statistically significant difference between the intervention and comparator at 9 months after exposure when QALYs and all categories of health care consumption were analyzed jointly (*P*=.46). When analyzed separately, there was a significant increase in the number of consultations made in private mental health care (*P*=.03). The intervention was associated with 0.0065 (95% CI −0.0219 to 0.0349) QALYs gained per user and an increment in costs of SEK −46,359 (95% CI −111,696 to 18,977; US $–4368, 95% CI −10,525 to 1788) per user compared to no guided self-management. Cost savings were due to fewer consultations and care days per user in public health care (−5.50, 95% CI −14.83 to 3.83). The intervention had a 62% probability of both gaining QALYs and saving costs, and the probability that it would be cost-effective remained constant at 92% over our threshold range. The total expected value of perfect information was SEK 5.4 million (US $510,480) and was largely attributable to statistical uncertainty in incremental costs.

**Conclusions:**

The use of a mobile app for self-management of posttraumatic stress was found to be cost-effective in a Swedish setting. A value-of-information analysis suggests that current research is sufficient to support the use of the app in Swedish practice from a cost-effectiveness perspective. However, to support its adoption in other settings or the potential of app-based interventions in general, stronger cost-effectiveness evidence is required.

**Trial Registration:**

ClinicalTrials.gov NCT04094922; https://clinicaltrials.gov/ct2/show/NCT04094922

## Introduction

### Background

Posttraumatic stress encompasses a range of emotional, cognitive, behavioral, and physiological reactions that stem from a single or multiple traumatic events. If severe and debilitating, the reactions may fulfill criteria for posttraumatic stress disorder (PTSD). Symptoms include intrusive thoughts, memories, or dreams of the traumatic event; efforts to avoid thoughts, feelings, sensations, or reminders of the event; distorted, more negative, vigilant, or numbed mood, thoughts, and interpretations; and heightened physiological arousal and reactivity [1,2]. Exposure to traumatic events is common worldwide [3,4]. A substantial minority of those exposed develop long-lasting trauma-related distress [5] or PTSD, with a cross-national lifetime prevalence of 3.9% [6]. The negative effects of PTSD are compounded by an increased risk of somatic and psychiatric comorbidity such as mood disorders, substance and alcohol use disorders, and suicidality [7].

Despite the existence of recommended, efficient psychological and medical treatments for PTSD, many people who have experienced traumatic events do not seek or seek but do not receive professional help [6]. Reasons behind the treatment gap include lack of knowledge about the disorder and trauma-focused care; limited personal or organizational resources; or emotional barriers such as stigma, fear, or shame among both patients and clinicians [8-10].

The development and implementation of digital interventions and mobile apps for mental health have attracted interest from researchers, clinicians, and health care policymakers as a potential medium through which to provide accessible, high-quality, and affordable options at different levels of care [11]. One of the initiatives taken is the development of apps with the aim to prevent trauma-related complications and reduce posttraumatic stress symptoms [12]. Digital tools hold promise as efficient means of serving a large portion of the global population and reducing waitlists while lowering mental health care expenditures [13]. In particular, digital interventions administered through self-management may be cost-effective or low-cost options for providing trauma-focused psychoeducation and care [14-17].

An example of a digital self-management intervention is the mobile app PTSD Coach [12,18,19]. The increased use of smartphones and high prevalence of PTSD among military veterans inspired its development by the US Department of Veterans Affairs National Center for PTSD and the US Department of Defense National Center for Telehealth and Technology [20]. The app has since been freely and publicly available as a resource for veterans, service members, and civilians affected by traumatic events [20]. The purpose of PTSD Coach is to help individuals better understand and manage their trauma-related symptoms. A model of openly sharing the source code has resulted in versions of the app being developed worldwide, including in North America [12,18,20], Europe [12,21-23], Oceania [12], and Africa [24,25].

However, the promises of self-management apps for mental health are not without some uncertainties. In controlled comparisons, their efficacy for reducing posttraumatic stress is unclear [16,26-28], and some question whether apps should be used independently of clinical support and warn against potential threats to confidentiality or negative effects [26,29,30]. Furthermore, because digital interventions are cheaper at the point of delivery than face-to-face treatment, it has been claimed that they are cost-effective despite a lack of evidence to support such claims [31]. Not only is economic evaluation necessary to determine whether cost savings or benefits to users are large enough to justify app development and maintenance costs, but for a self-management app, it is also ambiguous a priori whether its adoption will lead to a reduction or increase among users in use of face-to-face care. Systematic reviews have demonstrated limited cost-effectiveness evidence on self-management [32] and internet- or mobile-based interventions for mental health [31], identifying only 1 economic evaluation of an app intervention for posttraumatic stress [33].

### Objectives and Context

In this study, we contributed to the cost-effectiveness evidence on internet-based interventions for posttraumatic stress by conducting an economic evaluation of the Swedish version of PTSD Coach [21,34], which has been evaluated with respect to trauma-related symptoms in a randomized controlled trial (RCT), where it was shown that having access to the app significantly reduced posttraumatic stress, depressive symptoms, and functional disability [34,35]. The lifetime prevalence of PTSD in Sweden has been estimated at 5.6% (3.6% for men and 7.4% for women) [36], which is comparable to the prevalence observed in other high-income countries [6]. Administrative health care records indicate that 0.7% of the Swedish working-age population received specialized care treatment for PTSD during the period from 2006 to 2016, with a similar overrepresentation of women [7].

## Methods

### Study Design

This was an economic evaluation from a payer (public health care sector) perspective that was conducted alongside an RCT [34] in which participants from a convenience sample recruited through social media were randomly assigned to be given either immediate access to PTSD Coach (intervention group) or delayed access at 3 months after randomization (waitlist group). All trial participants were surveyed using a web-based written questionnaire at baseline and at 3, 6, and 9 months of follow-up regarding functional disability and health care consumption. Treatment effects at 3 months after exposure were identified through the trial’s random assignment. At 6 and 9 months after exposure, there was no unexposed control group. Treatment effects at these horizons were identified under the assumption that the intervention’s short-term effects were the same in the waitlist group as in the intervention group (see (see the Results section for an illustration).

### Scope

Results were reported in agreement with the Consolidated Health Economic Evaluation Reporting Standards [37]. Results on efficacy, helpfulness, satisfaction, and negative effects and further details on the trial procedures have been reported elsewhere [34,35].

### Ethical Considerations

The regional ethical review board in Uppsala, Sweden, approved the trial procedures before data collection (reference 2018/319). The ethical review encompassed the purpose of this study. Participants provided written informed consent before inclusion in the trial. All study data were deidentified to protect confidentiality and participants’ privacy. Participation was compensated with gift cards for 2 movie tickets after follow-up. The trial was registered on ClinicalTrials.gov (NCT04094922).

### Participants

The trial included 179 participants (intervention group: n=89, 49.7%; waitlist group: n=90, 50.3%) who had been exposed to a potentially traumatic event in the previous 2 years and were experiencing mild to severe posttraumatic stress symptoms, assessed using the PTSD Checklist for the *Diagnostic and Statistical Manual of Mental Disorders, Fifth Edition* [38], and a cutoff score ≥10 points for minimal posttraumatic stress. Some participants (intervention group: 38/89, 43%; waitlist group: 39/90, 43%) had previously received psychological treatment or counseling related to a traumatic event at some point [34] or were currently on a stable dose of medication to manage their symptoms, but they were not included if they were currently undergoing other psychological treatments.

### Intervention

PTSD Coach provides psychoeducation about posttraumatic stress, prevalence, symptoms, and treatments; self-assessment of PTSD symptoms with historic ratings and automatic feedback; exercises for managing distress based on evidence-based treatments; and, contact information to support services within the app [18,20]. The comparator in our analyses was the absence of app-guided self-management.

### Health-Related Quality of Life and Health Care Consumption

Health-related quality of life (HRQoL) was measured based on self-reported functional disability using the 12-item version of the World Health Organization Disability Assessment Schedule (WHODAS) [39,40]. A validated mapping function between the WHODAS and the visual analogue scale [41] was used to combine responses to the separate WHODAS items into an index of HRQoL on a scale from 0 to 1.

Health care consumption was measured using a Swedish translation and adaptation (available online [42]) of the 14-item health care consumption section of the Treatment Inventory of Costs in Patients With Psychiatric Disorders (TiC-P) [43]. Participants were asked to provide details on the number of consultations or visits with different categories of health care staff (in the previous month or in the previous 3 months), the number of inpatient care days (in the previous 3 months), and the dose and frequency of medications taken (in the previous 3 months). Responses to a small number of TiC-P items were missing (48/8700, 0.55%). These observations were imputed by carrying the previous observation forward or, at baseline, the next observation backward.

### Costs

Pharmaceutical consumption (TiC-P item 14) was converted to monetary costs using drug prices collected on December 18, 2023, from the Swedish medicine compendium Fass (Table S1 in Multimedia Appendix 1). Costs were categorized as either related (drugs indicated for PTSD, depression, anxiety, sleeping problems, bipolar disorder, or attention-deficit/hyperactivity disorder) or unrelated. We assumed that all pharmaceutical consumption was financed by the Swedish Pharmaceutical Benefit (in reality, purchases up to SEK 6381 [US $601] per person and year would have been partially paid for out of pocket before being fully covered through a stepwise discount scheme). The monetary cost of health care use was calculated using unit costs retrieved from the Swedish Association of Local Authorities and Regions cost-per-patient database for 2022 (Table S2 in Multimedia Appendix 1) and inflated to the 2023 price level using a health care consumer price index from Statistics Sweden. In the main analysis, unit costs were applied only to TiC-P items pertaining to publicly funded health care.

From public launch at the end of 2021 to the end of 2023, the app was downloaded 1976 times. During this period, maintenance costs were approximately SEK 24,000 (US $2261). Therefore, the cost per user was assumed to be SEK 12 (ie, SEK 24,000/1976; US $1.13).

### Analyses

#### Statistical Analysis

For each outcome (HRQoL, 13 types of health care use, and 2 types of drug costs), the treatment effect at 3 months after exposure was estimated using an intention-to-treat approach as the difference in means between the intervention and waitlist groups. The treatment effect at 6 months after exposure was estimated as a difference in means between the intervention and a counterfactual comparator group. The comparator mean was calculated by subtracting the estimated 3-month treatment effect from the waitlist group mean at 6 months of follow-up, which, for the waitlist group, corresponded to 3 months after exposure because of the delay in access. The 9-month treatment effect was similarly estimated by first subtracting the estimated 6-month treatment effect from the waitlist mean at 9 months of follow-up. The cumulative effect on each outcome at 9 months after exposure was then derived as the area under the curve (AUC) formed by the treatment effects and discounted at a rate of 3% per year [44]. Estimation was done via linear regression, and all outcome equations were estimated jointly to allow for correlation between the outcomes and joint hypothesis testing across equations. Hypothesis tests were conducted for significance at a 5% level using heteroskedasticity-consistent methods with clustering at the participant level [45]. All analyses were conducted in R (version 4.4.1; R Foundation for Statistical Computing) [46]. Further methodological details are provided in Multimedia Appendix 1.

#### Cost-Effectiveness Analysis

The incremental effectiveness (quality-adjusted life years [QALYs] gained or lost) and incremental costs (cost savings or increases) with the intervention versus the comparator were assessed against a cost-effectiveness threshold that represented the cost at which the public health care sector could be expected to produce a QALY through other activities. With a cost-increasing intervention, these activities would be forgone [47], whereas with a cost-saving intervention, additional resources would instead be freed up for these activities [48]. The intervention was considered cost-effective if the number of QALYs gained exceeded the number of QALYs forgone. We used SEK 500,000 (US $47,113) per QALY as our base-case threshold and considered a range of up to SEK 1 million (US $94,225) per QALY to reflect thresholds that could be relevant in the context of Swedish health care priority setting [49,50].

A probabilistic sensitivity analysis was conducted to summarize the uncertainty in our cost-effectiveness results. Because the treatment effect estimators are approximately normally distributed according to the central limit theorem and incremental effectiveness and incremental costs for the intervention versus the comparator are linear combinations of the treatment effects, we assumed that incremental effectiveness and incremental costs followed a bivariate normal distribution [51,52]. This distribution was illustrated on the cost-effectiveness plane, and the probability of the intervention being cost-effective over our range of cost-effectiveness thresholds was calculated through integration without the need to conduct Monte Carlo simulations [52].

#### Value of Information Analysis

A probability of cost-effectiveness exceeding 50% indicates that an intervention is more likely than not to be an efficient use of health care resources, but even when the probability of cost-effectiveness is very high (or very low), the evidence used for a cost-effectiveness analysis can be associated with a substantial amount of uncertainty. The appropriateness of adoption or rejection of a new intervention based on current evidence can be assessed through value of information analysis [53].

Therefore, to further support decision-making about the use of PTSD Coach in a Swedish context, we estimated the expected value of perfect information (EVPI) [54] over our range of cost-effectiveness thresholds. The EVPI was the expected monetary value of removing all decision uncertainty, and as such, it constituted an upper limit to the cost of further research, at which point it would be better to decide on adoption or rejection based on current evidence. To analyze the sources of decision uncertainty, we also estimated the EVPI on incremental effectiveness and incremental costs separately (expected value of partial perfect information; EVPPI). Both the EVPI per user and the EVPPI per user were derived analytically from the joint distribution of incremental effectiveness and incremental costs using the unit normal loss integral method [55]. Total EVPI and EVPPI were calculated assuming 988 users per year and a time horizon of 5 years at a 3% discount rate [44].

#### Sensitivity Analyses

We conducted a number of sensitivity analyses with respect to estimation method, attrition, missing data, private health care consumption, number of potential users, and app cost. First, to account for potential baseline imbalances, treatment effects were estimated by difference in differences [56] and with adjustment for outcomes at baseline [57]. Second, to consider a scenario in which HRQoL and health care consumption would have remained unchanged in the absence of the intervention, treatment effects were also estimated through a pre-post analysis for the intervention group. Third, bias from attrition was investigated using complete cases only and through a best-case and worst-case scenario analysis where we imputed missing observations with either the 5th or 95th percentile of outcomes; 179, 149, 134, and 118 responses were collected at baseline and 3, 6, and 9 months, respectively. A total of 58.1% (104/179) of the participants responded at all points of follow-up. Fourth, to investigate whether results were sensitive to the choice of approach for imputing missing TiC-P responses, we used multiple imputation with predictive mean matching [58]. Fifth, unit costs were applied to TiC-P items pertaining to private care (3, 5, 10, and 11) to assess how the cost-effectiveness of the intervention would have been affected had all health care consumption been publicly funded. Sixth, an estimate of the incidence of PTSD treatment [7] was used for a scenario with a higher number of future users (3586 per year). Finally, the cost of development and maintenance for the Swedish version of the app from 2018 until public launch (SEK 194,855 [US $18,360]) was used to estimate an upper bound for the cost per user at SEK 111 ([SEK 194,855 +SEK 24,000]/1976; US $10.46).

## Results

### Baseline Characteristics

The mean age among participants was 42.8 (SD 10.9; range 18-68) years, and there was a notable underrepresentation of men (11/179, 6.1%) compared to the general population. In total, 61.5% (110/179) of participants reported at least one visit or consultation in some form of care during the 3 months leading up to the trial, and approximately one-third (63/179, 35.2%) had used some kind of psychiatric medication at least once in the previous 3 months. A small number of participants had visited a psychiatrist, psychologist, or psychotherapist in either public (13/179, 7.3%) or private (8/179, 4.5%) care in the previous month. Use of health care appeared to be slightly more common in the intervention group when health care consumption items were considered separately (Table 1), but the number of participants who reported some use of care was similar across groups (waitlist group: 54/90, 60%; intervention group: 56/89, 63%). Overall, differences in outcomes at baseline between the intervention and waitlist groups were small and not apparently systematic.

**Table 1:** Age, sex, health-related quality of life (HRQoL), and health care consumption (HC) at baseline.

Characteristic		Waitlist group (n=90)	Intervention group (n=89)
Age (y), mean (SD; range)		42.2 (11.2; 18-68)	43.4 (10.6; 20-67)
Men, n (%)		4 (4)	7 (8)
HRQoL (index; 0-1), mean (SD)		0.5701 (0.0892)	0.5581 (0.0874)
**Past-month HC^a^, n (%)**			
	1. General practitioner	30 (33)	30 (34)
	2. Health care professional in home care	2 (2)	2 (2)
	3. Mental health care, private^b^	3 (3)	5 (6)
	4. Mental health care, public^c^	6 (7)	7 (8)
	5. Physician in occupational health care	1 (1)	1 (1)
**Past–month HC cost^d^, mean (SD)**			
	Public health care^e^	3196 (4802)	3779 (5833)
	All health care^f^	3500 (5043)	4575 (7199)
**Past–3-month HC, n (%)**			
	6. Specialist physician^g^	8 (9)	11 (12)
	7. Paramedical professional^h^	20 (22)	21 (24)
	8. Social worker or counselor	11 (12)	12 (13)
	9. Clinic for alcohol or drug use	1 (1)	1 (1)
	10. Alternative medicine practitioner	3 (3)	8 (9)
	11. Self-help group	2 (2)	6 (7)
	12. Psychiatric day care program	1 (1)	0 (0)
	13. Inpatient care days	4 (4)	8 (9)
**Past–3-month HC cost^d^, mean (SD)**			
	Public health care^e^	11,933 (53,229)	10,235 (33,680)
	All health care^f^	13,314 (53,883)	12,008 (33,980)
**Past–3-month medication use, n (%)**			
	Related drugs	33 (37)	30 (34)
	Unrelated drugs	32 (36)	36 (40)
**Past–3-month drug cost^d^, mean (SD)**			
	Related drugs	168 (694)	244 (798)
	Unrelated drugs	716 (3327)	282 (645)

^a^Any visit or consultation with the specified type of health care professional. The leading number indicates which Treatment Inventory of Costs in Patients With Psychiatric Disorders (TiC-P) item the visits and consultations pertain to.

^b^Psychiatrist, psychologist, or psychotherapist in private care, paid for out of pocket.

^c^Psychiatrist, psychologist, or psychotherapist in publicly funded outpatient care.

^d^SEK, 2023 price level; SEK 1=US $0.094, average exchange rate in 2023.

^e^Items 1,2, and 4 for past–month health care consumption and items 6-9 and 12-13 for past–3-month health care consumption.

^f^Items 1-5 for past–month health care consumption and items 6-13 for past–3-month health care consumption.

^g^Specialist physician in hospital-based outpatient care.

^h^For example, physiotherapist, speech therapist, massage therapist, or occupational therapist.

### Effects on HRQoL, Health Care Consumption, and Costs

**Figure 1:**
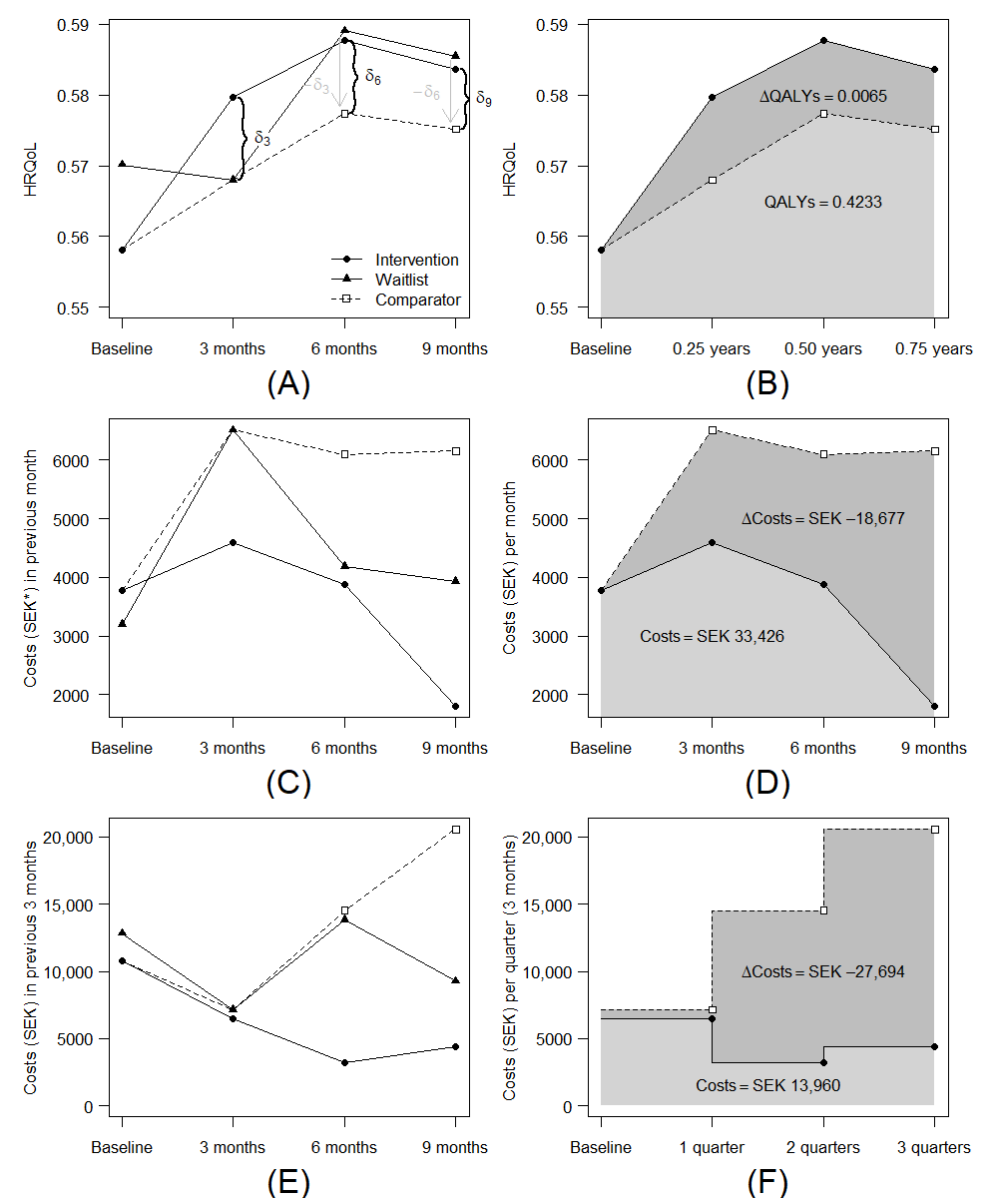
Mean health-related quality of life (HRQoL) or costs (SEK; *SEK 1=US $0.09) at 3, 6, and 9 months of follow-up and quality-adjusted life years (QALYs) or cumulative costs with the intervention versus the comparator at 9 months after exposure―(A) HRQoL with an illustration of how treatment effects (δ) and comparator means at 6 and 9 months were derived; (B) QALYs; (C) and (D) costs for past–month health care consumption items; (E) and (F) costs for past–3-month health care consumption items.

Table 2 reports the estimated cumulative effects of the intervention over the study period. There was no statistically significant difference between the intervention and comparator at 9 months after exposure when all outcomes (HRQoL and multiple types of health care consumption) were analyzed jointly (*P*=.46). When analyzed separately, there was an increase in the number of consultations with a psychiatrist, psychologist, or psychotherapist for which the participants paid out of pocket (3.61, 95% CI 0.35-6.87) but in no other outcomes. The point estimates indicated 0.0065 (95% CI −0.0219 to 0.0349) QALYs gained with the intervention versus the comparator and fewer publicly funded consultations, visits, and inpatient care days (−5.50, 95% CI −14.83 to 3.83). The total change in public health care costs from the intervention was SEK −46,359 (95% CI −111,696 to 18,977; US $–4368, 95% CI −10,525 to 1788). Figure 1 plots the intervention, waitlist, and comparator means in each follow-up period, as well as the AUC for HRQoL and aggregated costs (note that past-month and past–3-month costs could not be aggregated because the AUC was calculated differently for 1- and 3-month costs).

**Table 2:** The effect of PTSD Coach versus no app-guided self-management on quality-adjusted life years (QALYs) and cumulative health care consumption at 9 months after exposure.

Outcome			Effect (95% CI)
**Incremental effectiveness and costs**			
	QALYs	0.0065 (−0.0219 to 0.0349)	
	Public health care costs^a^	−46,359 (−111,696 to 18,977)	
**Number of health care consultations or visits**			
	General practitioner	−1.96 (−6.82 to 2.90)	
	Health care professional in home care	−0.91 (−2.92 to 1.10)	
	Mental health care, private^b^	3.61 (0.35 to 6.87)	
	Mental health care, public^c^	−1.07 (−3.74 to 1.60)	
	Physician in occupational health care	−0.68 (−2.81 to 1.45)	
	Specialist physician^d^	−0.55 (−1.97 to 0.86)	
	Paramedical professional^e^	0.61 (−1.42 to 2.64)	
	Social worker or counselor	−0.04 (−1.26 to 1.17)	
	Clinic for alcohol or drug use	−0.38 (−1.13 to 0.37)	
	Alternative medicine practitioner	1.13 (−0.01 to 2.27)	
	Self-help group	2.22 (−1.66 to 6.10)	
	Psychiatric day care program	−0.26 (−0.63 to 0.12)	
	Inpatient care days	−0.93 (−2.73 to 0.87)	
**Drug costs^a^**			
	Related drugs	−189 (−1686 to 1309)	
	Unrelated drugs	−4161 (−9811 to 1490)	

^a^SEK, 2023 price level; SEK 1=US $0.094, average exchange rate in 2023.

^b^Psychiatrist, psychologist, or psychotherapist in private care, paid for out of pocket.

^c^Psychiatrist, psychologist, or psychotherapist in publicly funded outpatient care.

^d^Specialist physician in hospital-based outpatient care.

^e^For example, physiotherapist, speech therapist, massage therapist, or occupational therapist.

### Cost-Effectiveness

**Figure 2:**
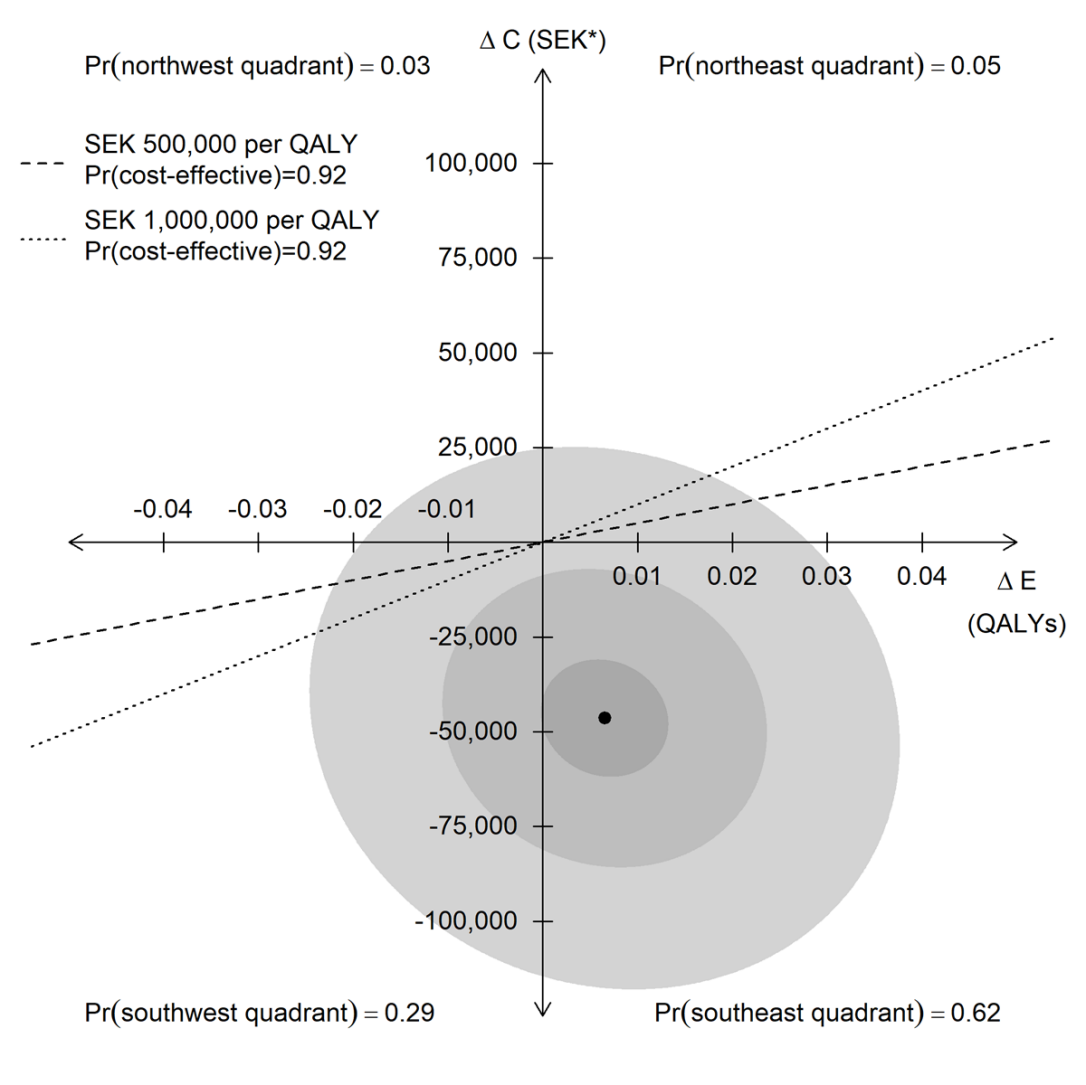
The incremental effectiveness (ΔE) and incremental costs (ΔC; *SEK 1=US $0.09) of PTSD Coach versus no app-guided self-management and the probability (Pr) of the intervention being cost-effective. The ellipses represent 90%, 50%, and 10% confidence regions for the joint distribution of incremental effectiveness and costs.

The estimated joint distribution of incremental effectiveness and incremental costs is plotted on the cost-effectiveness plane in Figure 2. Although there were no significant differences between the intervention and comparator in terms of QALYs (*P*=.65) or costs (*P*=.16), there was a 62% probability that the intervention dominated the comparator (ie, that it was both more effective and less costly). At a cost-effectiveness threshold of SEK 500,000 (US $47,113) per QALY, the intervention had a 92% probability of being cost-effective. The probability of cost-effectiveness was unaffected by lowering the threshold to SEK 1 (US $0.09) per QALY or raising it to SEK 1 million (US $94,225) per QALY.

### Value of Information

**Figure 3:**
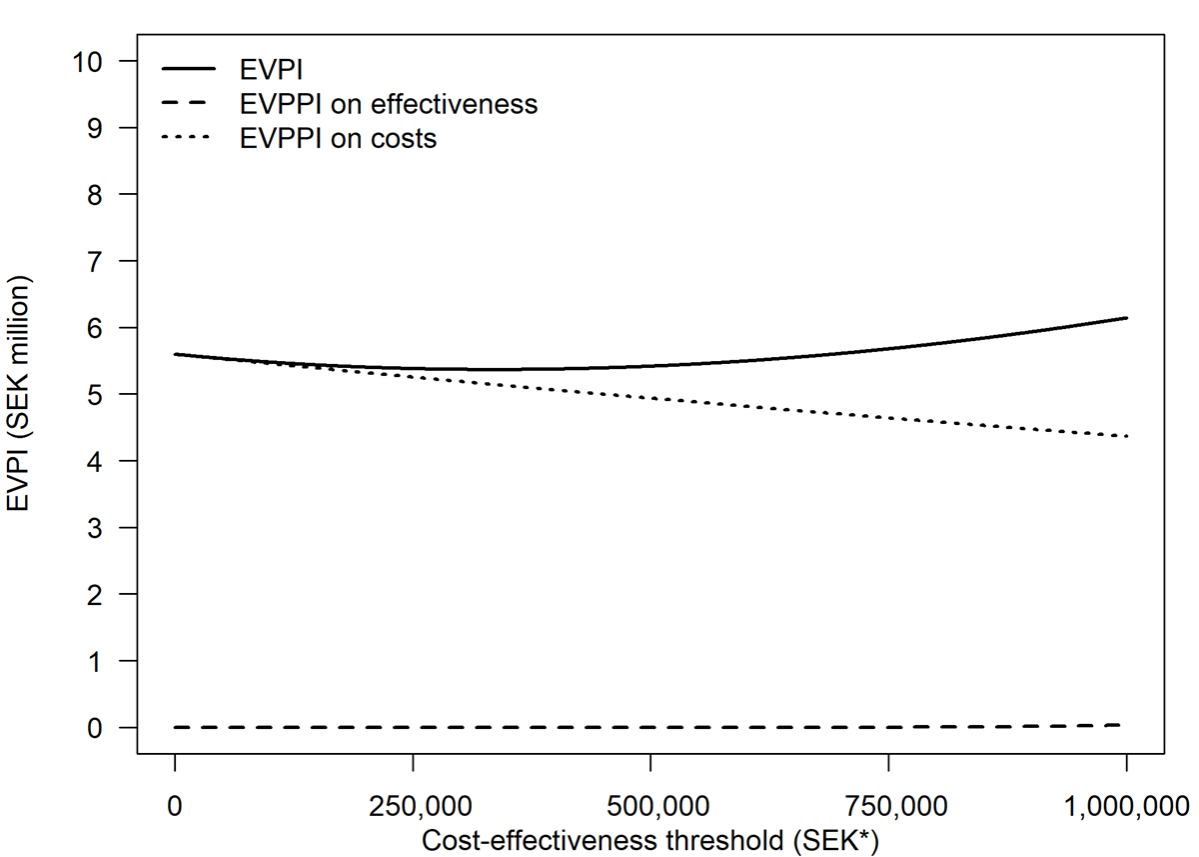
Expected value of perfect information (EVPI) and expected value of partial perfect information (EVPPI) on incremental effectiveness and costs. *SEK 1=US $0.09.

Figure 3 plots the results from our value of information analysis over the threshold range. At a cost-effectiveness threshold of SEK 500,000 (US $47,113) per QALY, the EVPI was SEK 5,417,642 (US $510,480). The expected value of having perfect information on the intervention’s effect on health care consumption alone was SEK 4,937,617 (US $465,249), whereas the expected value of knowing the number of QALYs gained or lost with certainty was only SEK 12 (US $1.13).

### Sensitivity Analyses

The intervention dominated the comparator across all scenarios considered in our sensitivity analyses, with the probability of cost-effectiveness never falling below 75% (Table 3). Results on value of information from these analyses are reported in Multimedia Appendix 2.

**Table 3:** The cost-effectiveness of PTSD Coach versus no app-guided self-management in sensitivity analyses.

Analysis			ΔEffectiveness (95% CI)^a^		ΔCosts (95% CI)^b^		Pr CE^c^		Pr SE^d^
Main analysis			0.0065 (−0.0219 to 0.0349)		−46,359 (−111,696 to 18,977)		0.92		0.62
**Estimation**									
	Difference in differences	0.0198 (−0.0024 to 0.0420)		−41,948 (−135,781 to 51,884)		0.86		0.78	
	Baseline adjustment	0.0137 (−0.0046 to 0.0320)		−45,217 (−109,280 to 18,846)		0.94		0.86	
	Pre-post	0.0158 (0.0069 to 0.0246)		−18,170 (−39,265 to 2924)		0.99		0.95	
**Attrition^e^**									
	Complete cases	0.0058 (−0.0283 to 0.0399)		−48,170 (−137,315 to 40,975)		0.86		0.55	
	Negative attrition	0.0003 (−0.0283 to 0.0290)		−25,532 (−91,587 to 40,523)		0.75		0.45	
	Positive attrition	0.0097 (−0.0167 to 0.0361)		−38,651 (−92,521 to 15,219)		0.93		0.71	
**Miscellaneous**									
	Multiple imputation	0.0065 (−0.0219 to 0.0349)		−46,274 (−111,133 to 18,585)		0.92		0.62	
	Including private care	0.0065 (−0.0219 to 0.0349)		−24,554 (−94,994 to 45,886)		0.77		0.52	
	More users	0.0065 (−0.0219 to 0.0349)		−46,359 (−111,696 to 18,977)		0.92		0.62	
	Higher app cost	0.0065 (−0.0219 to 0.0349)		−46,260 (−111,597 to 19,076)		0.92		0.62	

^a^Incremental effectiveness, quality-adjusted life years.

^b^Incremental costs, SEK, 2023 price level; SEK 1=US $0.094, average exchange rate in 2023.

^c^Probability of the intervention being cost-effective at a cost-effectiveness threshold of SEK 500,000 (US $47,113) per quality-adjusted life year.

^d^Probability of the intervention being dominant (in the southeast quadrant of the cost–effectiveness-plane).

^e^In the negative attrition scenario, the 95th percentile of health care consumption and the 5th percentile of health-related quality of life were used to impute missing observations for participants lost to follow-up; the percentiles were reversed in the positive attrition scenario.

## Discussion

### Principal Findings

We found that PTSD Coach had a 92% probability of being cost-effective and a 62% probability of both improving HRQoL and reducing health care costs. Because the intervention dominated the comparator, it was not necessary or meaningful to calculate an incremental cost-effectiveness ratio [59], which is the typical metric reported by economic evaluations to inform judgments about whether new interventions are effective enough to justify higher costs. We also found that the probability of cost-effectiveness was insensitive to the choice of cost-effectiveness threshold (up to at least SEK 1 million [US $94,225] per QALY), which removed another difficulty usually involved in interpreting cost-effectiveness evidence [60].

Our value of information analysis provided a framework through which to judge whether the probability of cost-effectiveness was acceptable and help reconcile such an interpretation with the perhaps seemingly contradictory finding that there was no significant difference between the intervention and comparator [61]. The EVPI, estimated at SEK 5.4 million (US $510,480), constitutes an upper limit to how much we should be willing to spend on further research to become more certain about a decision to provide (or not to provide) continued access to the app. Considering the costs of conducting a trial and that a trial could never provide perfect information, it seems unlikely that further research would be worthwhile for the sole purpose of informing this particular decision. This reflects the common-sense conclusion that the optimal amount of time spent preparing for a decision must be determined by how important the decision is. Had the magnitude of health gains or cost savings been greater, the stakes would have been higher, and more research could have been motivated even in a situation in which the estimates had been statistically significant. Similarly, the value of having stronger evidence would increase with the number of app users. Therefore, current evidence appears sufficient to support the cost-effectiveness of PTSD Coach in a Swedish context with use at or moderately over its current level, but this conclusion should not be generalized to a decision about large-scale adoption in another context.

Furthermore, we found that the intervention’s effect on health care consumption was the most important determinant of its cost-effectiveness. App development costs, in comparison, had a negligible impact. One might be inclined to attribute this finding to the fact that translating and adapting an already existing app must be much less costly than developing a new one from scratch, but for the intervention to become cost ineffective, development costs for the Swedish version of the app would have had to be SEK 225 million (US $21,200,734); more than a thousand times as high as they actually were. The magnitude of incremental effectiveness was found to be relatively small as well. At our base-case threshold, the cost savings from the intervention would generate approximately 14 times as many QALYs in other parts of the health system as those gained by the users of the app themselves. We also found that health care consumption was the main source of decision uncertainty, further underscoring the importance of context. Our value of information analysis showed that there would be essentially no value to becoming more certain about the intervention’s effect on HRQoL (EVPPI=SEK 12 [US $1.13]) without first reducing uncertainty about its effects on health care consumption. If use of administrative records or register data could reduce noise in the measurement of costs and make estimates more precise, this is something that might be considered in future economic evaluations of internet- and mobile-based interventions, which to date have tended to rely on self-reported health care consumption [31].

Finally, our results were suggestive of cost savings being generated not so much through a reduction in health care consumption as through a shift in use from public health care to other forms of care, such as private therapy, participation in self-help groups, and alternative medicine. We speculate that access to PTSD Coach might have stimulated a sense of urgency among users in dealing with their symptoms and led them to seek prompt access to care through private options rather than waiting for treatment in public health care, which can take several months. However, the motivation to access methods of coping may have preceded access to the intervention. We did find that the intervention would have remained cost saving had all health care consumption been publicly funded because less costly visits and consultations were substituted for more costly forms of care (eg, physician visits and hospitalizations), but it is still relevant to note that the effects of the intervention might differ between settings and over time depending on users’ access to care.

### Implications for Practice

It is important to consider the role that PTSD Coach might play in the treatment setting for posttraumatic stress. Self-guided digital interventions differ from guided, in-person treatment in terms of customization; immediacy; and richness of verbal, visual, and emotional communication. Guidance during use of PTSD Coach has been proposed as a way to facilitate intervention effects and symptom improvement [24,62,63]. The app was not designed [64] to target known mechanisms of change (avoidance and cognitive distortions) for PTSD [65] but could be used as a low-impact complementary support to treatment [34,35] independently when treatment is not required, during waitlist to treatment, or in parallel with treatment (between sessions) together with a clinician. In these matters, the user’s personal preferences should be considered. Our study evaluated app-guided versus no app-guided self-management and does not support prioritizing the implementation of PTSD Coach over more efficacious medical, digital, and in-person treatment options for posttraumatic stress.

### Comparison to Previous Work

Cost-effectiveness evidence on app-based self-management for posttraumatic stress is scarce. A previous study on the cost-effectiveness of a self-help app targeting Syrian refugees with posttraumatic stress [33] found that the intervention was associated with insignificant reductions in both QALYs and costs and had a 20% probability of being cost-effective. Considering internet- and mobile-based interventions for mental health more generally, a systematic review [31] found that the interventions dominated the comparators in 13 out of the 30 economic evaluations included and that the interventions were cost-effective in almost 90% of cases. Our findings align with this previous evidence. Comparison with this previous research also reveals that we are not alone in reporting that an intervention is cost-effective but with substantial uncertainty. Only approximately one-third of the evaluations in the aforementioned systematic review found that the intervention had a probability of cost-effectiveness of at least 80% at conventional levels of the cost-effectiveness threshold. In comparison, the cost-effectiveness evidence reported in this study is relatively strong.

### Limitations

There are some limitations that must be considered when interpreting the results of this study. First, it should be noted that, although this study was based on an RCT that included assessments of health care consumption, the particular study design of the economic evaluation was detailed post hoc. As such, the results might be considered less reliable than had the original study protocol prespecified a detailed plan for the economic evaluation. This also meant that we were unable to administer a generic HRQoL instrument to participants. The practice of mapping disease- or condition-specific instruments to HRQoL weights is becoming more common, but generic instruments (eg, the EQ-5D) are still considered best practice in economic evaluation [66], which limits the possibility of comparing the magnitude of a QALY gain in this study to that of QALY gains in other studies.

Furthermore, it should again be stressed that the waitlist design of the RCT did not provide us with an unexposed control group for more than 3 months of follow-up. The assumption of equal treatment effects across groups is in one sense satisfied by the random assignment of treatment, but statistical uncertainty (and chance bias) in the estimated treatment effect at 3 months after exposure would nevertheless tend to multiply when used to construct the comparator means at 6 and 9 months, leading to less precise estimates than those that could have been attained had the control group remained unexposed beyond 3 months of follow-up.

Next, there is a risk that attrition over the 9-month study period led to a bias in our results. Our sensitivity analyses showed that attrition due to negative factors (poor HRQoL and high health care consumption) would have favored the intervention, whereas participants dropping out because of an improvement in their condition would have favored the comparator. However, our findings were not overturned even when missing outcomes were imputed under relatively extreme assumptions. Our sensitivity analyses accounting for random imbalances at baseline suggested that chance bias might have favored the comparator in terms of HRQoL, whereas incremental costs were largely unaffected by baseline adjustment (Table 3).

Another limitation to consider when interpreting our value of information analysis is that it could only incorporate statistical uncertainty from the trial results. This means that its exact interpretation is conditional on all other information, assumptions, and methodological choices being 100% certain. Other sources of uncertainty were, for example, our choice of estimation method, our approach to unit costing, the estimated number of future app users, or the assumptions made in our sensitivity analyses about participants who were lost to follow-up. Therefore, we caution against interpreting the EVPI as an exact number. Rather, it should be regarded as an indication that the value of acquiring more information is low.

Finally, the recruitment of participants through convenience sampling resulted in a very small share of male participants even in relation to the higher prevalence of PTSD among women [7,36]. This could limit the generalizability of our results, but in other important respects, such as educational level, employment, and marital status, the sample was comparable to the general population [34]. However, perhaps more importantly, the recruitment procedure meant that motivated participants may have applied to the trial in the hope of gaining access to a presumably effective intervention rather than being prompted to change. Therefore, some caution should be observed about generalizing our findings to a scenario of wider adoption where the average user might be less motivated. On the basis of the sample size, subgroup analyses were not judged to be feasible.

### Conclusions

The use of a mobile app for self-management of posttraumatic stress was found to be cost-effective in a Swedish setting. To support its adoption in another setting or the potential of app-based interventions in general, stronger cost-effectiveness evidence is required, but the low value of additional information suggests that further research would not be warranted from a cost-effectiveness perspective to inform a decision on whether to provide PTSD Coach in a Swedish context.

## Appendix

Multimedia Appendix 1 Methodological details and unit costs.

Multimedia Appendix 2 Value of information in sensitivity analyses of the cost-effectiveness of PTSD Coach versus no app-guided self-management.

Multimedia Appendix 3 Consolidated Health Economic Evaluation Reporting Standards 2022 checklist.

## Abbreviations

AUC: area under the curve
EVPI: expected value of perfect information
EVPPI: expected value of partial perfect information
HRQoL: health-related quality of life
PTSD: posttraumatic stress disorder
QALY: quality-adjusted life year
RCT: randomized controlled trial
TiC-P: Treatment Inventory of Costs in Patients With Psychiatric Disorders
WHODAS: World Health Organization Disability Assessment Schedule

## Data Availability

The datasets generated or analyzed during this study are available in the OSF repository [42].
