# Supplementary material for: Cost-Effectiveness of App-Guided Self-Management for Posttraumatic Stress: Trial-Based Economic Evaluation
Source: J Med Internet Res. 2025 Sep 18;27:e69426. doi: 10.2196/69426 (PMC12491891; doi:10.2196/69426)
Supplement: Multimedia Appendix 1 [file jmir_v27i1e69426_app1.pdf]

Supplemental material

Cost-effectiveness of app-guided self-management (PTSD Coach) for posttraumatic stress: trial-based economic evaluation

A.1 Methodological details

A.1.1 Estimation of treatment effects

For each outcome (Y_{it}), we estimated the following equation including indicator variables for intervention group (D_i), follow-up period (T_{3t}, T_{6t}, T_{9t}), and their interactions

$$Y_{it} = \alpha + \gamma_3 T_{3t} + \gamma_6 T_{6t} + \gamma_9 T_{9t} + \rho D_i + \beta_3 D_i T_{3t} + \beta_6 D_i T_{6t} + \beta_9 D_i T_{9t} + u_{it},$$

In our main analysis, the treatment effects (δ) at 3, 6, and 9 months were then estimated as

$$\hat{\delta}_3 = \hat{\rho} + \hat{\beta}_3$$
$$\hat{\delta}_6 = \hat{\delta}_3 + \hat{\rho} + \hat{\beta}_6$$
$$\hat{\delta}_9 = \hat{\delta}_6 + \hat{\rho} + \hat{\beta}_9$$

, where $\hat{\rho} + \hat{\beta}_t$ was the difference in means between the intervention group and waitlist group in follow-up period t (because D_i was randomly assigned, we can assume $E[\hat{\rho}] = 0$ but not $\hat{\rho} = 0$). In our sensitivity analyses, we consider two approaches to adjusting for baseline imbalances: (1) difference-in-differences, (2) including the outcome variable at baseline as a covariate. With differences-in-differences, treatment effects were instead estimated as

$$\hat{\delta}_3 = \hat{\beta}_3,$$
$$\hat{\delta}_6 = \hat{\delta}_3 + \hat{\beta}_6,$$
$$\hat{\delta}_9 = \hat{\delta}_6 + \hat{\beta}_9.$$

Figure S.1 illustrates how this relaxed the assumption that randomisation was successful in producing balance at baseline by introducing the new assumption that the intervention group would have experienced the same trend in outcomes as the waitlist group in absence of the intervention.

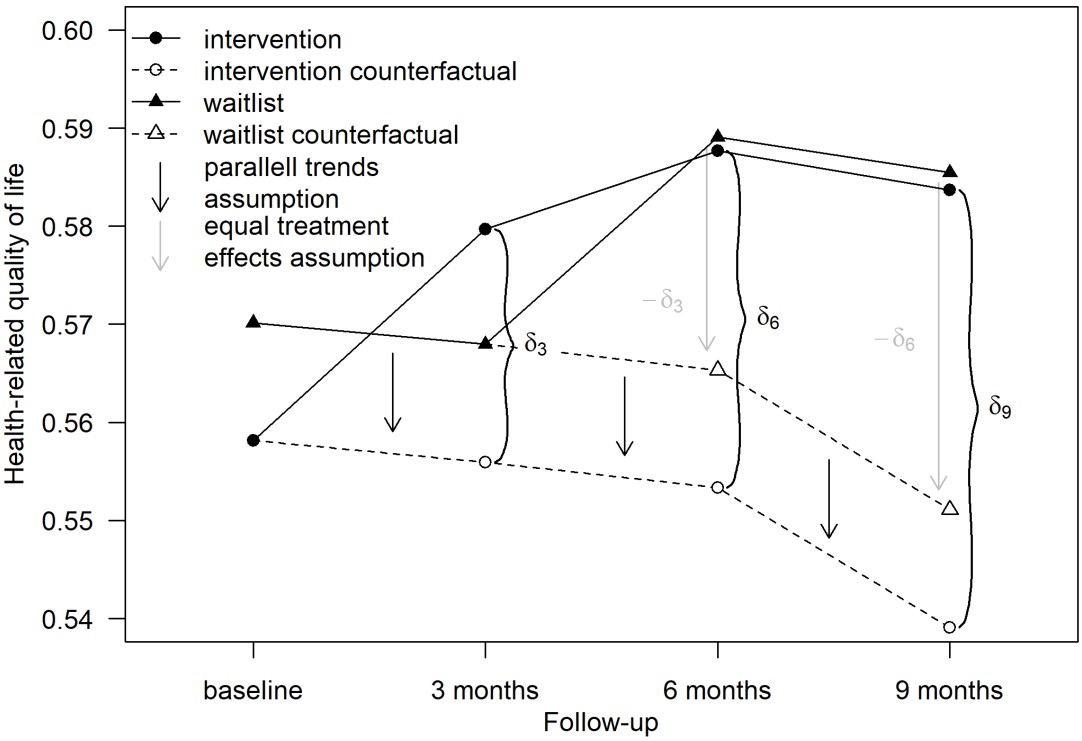

Figure S.1 Treatment effects identified by difference-in-differences

With explicit adjustment for outcomes at baseline, we re-estimated the following equation

$$Y_{it} = \alpha + \phi Y_{i0} + \gamma_6 T_{6t} + \gamma_9 T_{9t} + \beta_3 D_i T_{3t} + \beta_6 D_i T_{6t} + \beta_9 D_i T_{9t} + u_{it},$$

In this case, the treatment effects were also estimated as

$$\hat{\delta}_3 = \hat{\beta}_3,$$
$$\hat{\delta}_6 = \hat{\delta}_3 + \hat{\beta}_6,$$
$$\hat{\delta}_9 = \hat{\delta}_6 + \hat{\beta}_9.$$

Additionally, we considered a scenario in which HRQoL and healthcare consumption would have remained unchanged in the absence of the intervention. This was done by re-estimating the following equation for the intervention group alone

$$Y_{it} = \alpha \ + \beta_3 T_{3t} + \beta_6 T_{6t} + \beta_9 T_{9t} + u_{it}.$$

Here, the treatment effects were simply estimated as the pre-post difference ($\hat{\delta}_3 = \hat{\beta}_3$, $\hat{\delta}_6 = \hat{\beta}_6$, $\hat{\delta}_9 = \hat{\beta}_9$) as illustrated in Figure S.2.

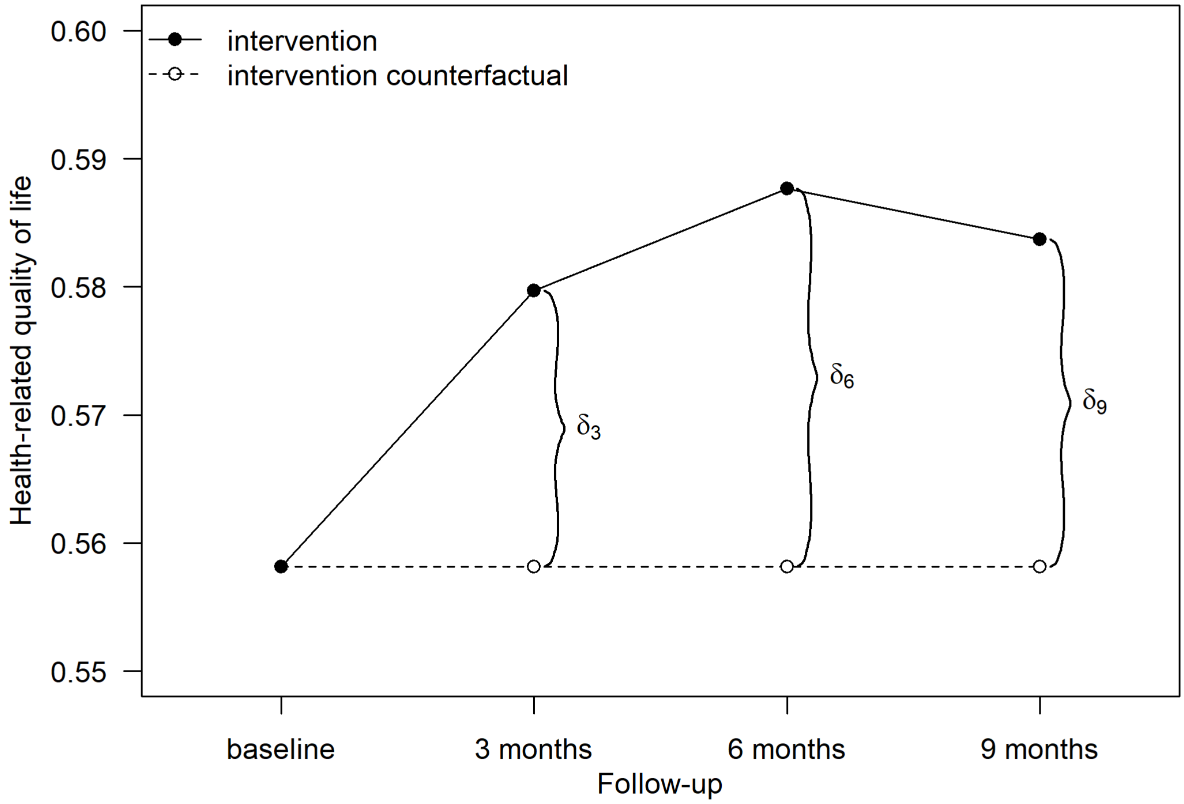

Figure S.2 Treatment effects from a pre-post analysis

A.1.2 Calculation of cumulative treatment effects

The cumulative treatment effect on each outcome was calculated as the area under the curve (AUC) formed by the treatment effects at 3, 6, and 9 months after exposure

$$AUC_3 = r_3 \lambda \hat{\delta}_3 \Delta t,$$
$$AUC_6 = AUC_3 + r_6(\hat{\delta}_3 + \lambda \hat{\theta}_6)\Delta t,$$
$$AUC_9 = AUC_6 + r_9(\hat{\delta}_3 + \hat{\theta}_6 + \lambda \hat{\theta}_9)\Delta t.$$

Here, $\hat{\theta}_6 = \hat{\delta}_6 - \hat{\delta}_3$ and $\hat{\theta}_9 = \hat{\delta}_9 - \hat{\delta}_6$ were incremental treatment effects; Δt was the duration of time between points of follow up in the unit relevant to the outcome (Δt = 0.25 years for HRQoL, Δt = 3 months for 1-month healthcare consumption, Δt = 1 quarter for 3-month healthcare consumption); r_t was a discount factor for a 3% per-annum rate, assuming the increments were realised in-between the points of follow up ($r_3 = 1.03^{-\left(\frac{1.5}{12}\right)}$, $r_6 = 1.03^{-\left(\frac{4.5}{12}\right)}$, $r_9 = 1.03^{-\left(\frac{7.5}{12}\right)}$); finally, for HRQoL and 1-month healthcare consumption, we assumed linear changes between the points of follow up ($\lambda = 0.5$). For example, AUC_3 (without discounting) for HRQoL would be a triangle with the area $0.25 \times \hat{\delta}_3 \times 0.5$ (see Figure S.3). For healthcare consumption outcomes measured over a full 3-month period, $\lambda = 1$.

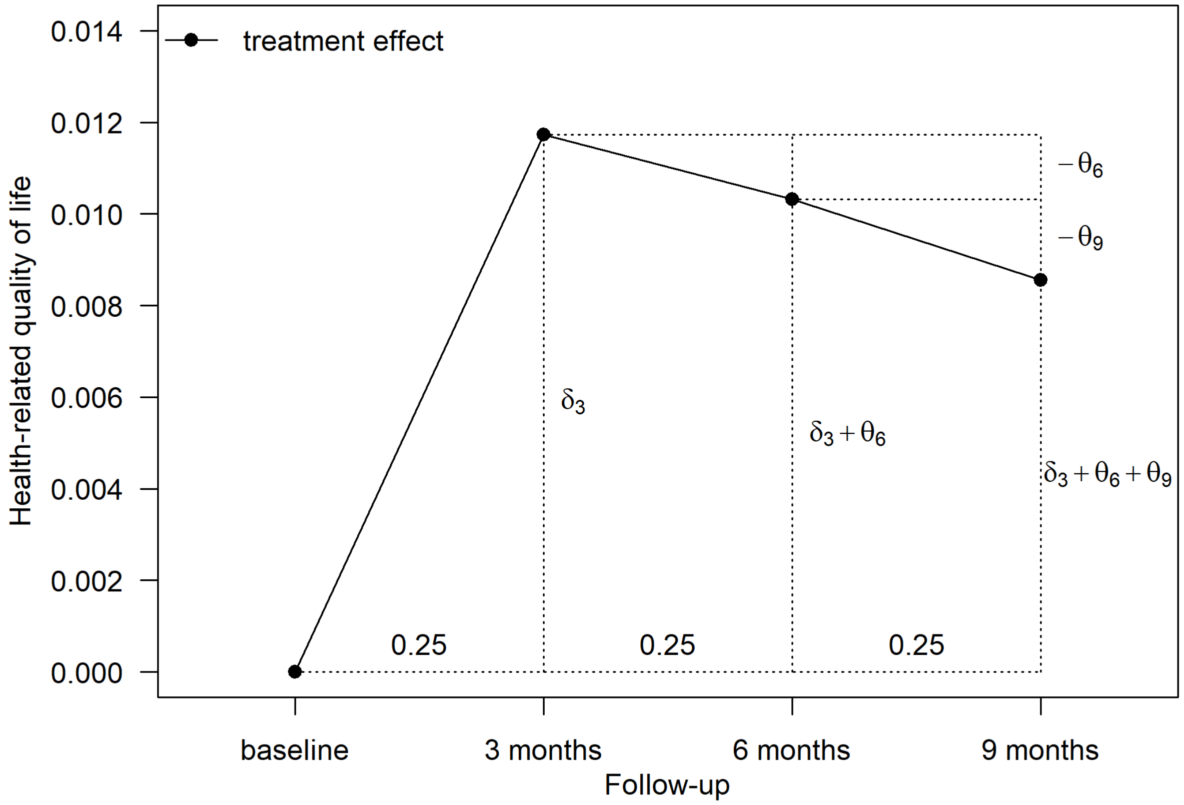

Figure S.3 Calculating the number of quality-adjusted life years (QALYs) gained as the area under the curve (AUC). $AUC_3 = 0.125\delta_3$; $AUC_6 = 0.375\delta_3 + 0.125\theta_6$; $AUC_9 = 0.625\delta_3 + 0.375\theta_6 + 0.125\theta_9$

A.2 Unit costs

Table S.1 Pharmaceutical prices

Product	Substance	Unit	PPU	R	URL
1	Acetylcysteine	100 mg	0.39		https://www.fass.se/LIF/product?userType=2&nplId=20041012000057
2	Acetylsalicylic acid	100 mg	1.17		https://www.fass.se/LIF/product?userType=2&nplId=20160315000028
3	Alendronic acid	100 mg	6.23		https://www.fass.se/LIF/product?userType=2&nplId=20051115000014
4	Alimemazine	100 mg	10.78	*	https://www.fass.se/LIF/product?userType=2&nplId=20170308000034
5	Alprazolam	100 mg	206.98	*	https://www.fass.se/LIF/product?userType=2&nplId=20100622000052
6	Amiloride + Hydrochlorothiazide	1 dose	1.04		https://www.fass.se/LIF/product?userType=2&nplId=19860626000017
7	Amitriptyline	100 mg	4.54	*	https://www.fass.se/LIF/product?userType=2&nplId=20130705000037
8	Amlodipine	100 mg	6.21		https://www.fass.se/LIF/product?userType=2&nplId=20131031000098
9	Apixaban	100 mg	435.98		https://www.fass.se/LIF/product?userType=2&nplId=20100501000012
10	Aripiprazole	100 mg	68.56	*	https://www.fass.se/LIF/product?userType=2&nplId=20140611000038
11	Atomoxetine	100 mg	8.51	*	https://www.fass.se/LIF/product?userType=2&nplId=20170622000086
12	Atovaquone + Proguanil	1 dose	10.90		https://www.fass.se/LIF/product?userType=2&nplId=20110531000081
13	Azelastine + Fluticasone propionate	1 dose	2.00		https://www.fass.se/LIF/product?userType=2&nplId=20111103000010
14	Beclometasone + Formoterol	1 dose	3.53		https://www.fass.se/LIF/product?userType=2&nplId=20071108000050
15	Bendroflumethiazide	100 mg	27.89		https://www.fass.se/LIF/product?userType=2&nplId=20130409000142
16	Bendroflumethiazide + Potassium chloride	1 dose	1.75		https://www.fass.se/LIF/product?userType=2&nplId=20090106000022
17	Betamethasone	100 mg	230.99		https://www.fass.se/LIF/product?userType=2&nplId=20171204000074
18	Bisoprolol	100 mg	15.85		https://www.fass.se/LIF/product?userType=2&nplId=20150501000154
19	Budesonide	1 dose	1.19		https://www.fass.se/LIF/product?userType=2&nplId=20040607000182
20	Budesonide	1 dose	1.27		https://www.fass.se/LIF/product?userType=2&nplId=20040607005699
21	Budesonide	1 dose	1.29		https://www.fass.se/LIF/product?userType=2&nplId=19881209000118
22	Budesonide + Formoterol	1 dose	3.78		https://www.fass.se/LIF/product?userType=2&nplId=20141001000010
23	Budesonide + Formoterol	1 dose	3.14		https://www.fass.se/LIF/product?userType=2&nplId=20150416000010
24	Budesonide + Formoterol	1 dose	2.87		https://www.fass.se/LIF/product?userType=2&nplId=20130411000116
25	Buprenorphine	100 mg	169.68		https://www.fass.se/LIF/product?userType=2&nplId=20090923000021
26	Buprenorphine	1 dose	1,008.93		https://www.fass.se/LIF/product?userType=2&nplId=20170727000042
27	Bupropion	100 mg	2.84	*	https://www.fass.se/LIF/product?userType=2&nplId=20131012000017
28	Buspirone	100 mg	24.05	*	https://www.fass.se/LIF/product?userType=2&nplId=20101104000102
29	Calcium carbonate + Cholecalciferol	1 dose	1.37		https://www.fass.se/LIF/product?userType=2&nplId=20200408000020
30	Carbomer	1 dose	86.00		https://www.fass.se/LIF/product?userType=2&nplId=20001215000223
31	Celecoxib	100 mg	1.63		https://www.fass.se/LIF/product?userType=2&nplId=19991203000035
32	Cetirizine	100 mg	10.85		https://www.fass.se/LIF/product?userType=2&nplId=20120214000024
33	Chlorzoxazone	100 mg	0.67		https://www.fass.se/LIF/product?userType=2&nplId=20181221000056
34	Cholecalciferol	1 dose	0.89		https://www.fass.se/LIF/product?userType=2&nplId=20170705000026
35	Citalopram	100 mg	5.96	*	https://www.fass.se/LIF/product?userType=2&nplId=20040607001585
36	Clemastine	100 mg	138.93		https://www.fass.se/LIF/product?userType=2&nplId=19690529000018
37	Clindamycin	100 mg	1.53		https://www.fass.se/LIF/product?userType=2&nplId=20110705000046
38	Clobetasol propionate	1 dose	68.66		https://www.fass.se/LIF/product?userType=2&nplId=19760121000034
39	Clopidogrel	100 mg	0.75		https://www.fass.se/LIF/product?userType=2&nplId=20120927000038
40	Cromoglicic acid	1 dose	3.46		https://www.fass.se/LIF/product?userType=2&nplId=19921204000048
41	Cyanocobalamin	100 mg	102.49		https://www.fass.se/LIF/product?userType=2&nplId=20010601000327
42	Dalteparin	1 dose	38.46		https://www.fass.se/LIF/product?userType=2&nplId=19880318000057
43	Desloratadine	100 mg	63.34		https://www.fass.se/LIF/product?userType=2&nplId=20190131000087
44	Dextroamphetamine	100 mg	157.25	*	https://www.fass.se/LIF/product?userType=2&nplId=20110721000068
45	Dextroamphetamine	100 mg	81.64	*	https://www.fass.se/LIF/product?userType=2&nplId=20111213000092
46	Diazepam	100 mg	20.09	*	https://www.fass.se/LIF/product?userType=2&nplId=20171221000187
47	Diclofenac	100 mg	3.04		https://www.fass.se/LIF/product?userType=2&nplId=20090728000028
48	Diclofenac	100 mg	3.01		https://www.fass.se/LIF/product?userType=2&nplId=20040916002587
49	Diltiazem	1 dose	342.43		https://www.fass.se/LIF/product?userType=2&nplId=20091026000635
50	Doxycycline	100 mg	4.30		https://www.fass.se/LIF/product?userType=2&nplId=19930319000041
51	Duloxetine	100 mg	2.51	*	https://www.fass.se/LIF/product?userType=2&nplId=20140816000055
52	Ebastine	100 mg	38.44		https://www.fass.se/LIF/product?userType=2&nplId=19950524000018
53	Enalapril	100 mg	3.84		https://www.fass.se/LIF/product?userType=2&nplId=20041208000038
54	Erenumab	1 dose	3,350.32		https://www.fass.se/LIF/product?userType=2&nplId=20170524000047
55	Escitalopram	100 mg	10.74	*	https://www.fass.se/LIF/product?userType=2&nplId=20140506000075
56	Esomeprazole	100 mg	10.21		https://www.fass.se/LIF/product?userType=2&nplId=20100325000038
57	Estradiol	100 mg	97.54		https://www.fass.se/LIF/product?userType=2&nplId=19700130000021
58	Estradiol	1 dose	16.16		https://www.fass.se/LIF/product?userType=2&nplId=20011130000277
59	Estriol	1 dose	3.61		https://www.fass.se/LIF/product?userType=2&nplId=19840203000050
60	Fentanyl	1 dose	23.11		https://www.fass.se/LIF/product?userType=2&nplId=20040916000811
61	Fexofenadine	100 mg	1.58		https://www.fass.se/LIF/product?userType=2&nplId=19970910000064
62	Fluoxetine	100 mg	8.42	*	https://www.fass.se/LIF/product?userType=2&nplId=20001020000036
63	Fluticasone furoate + Vilanterol	1 dose	11.28		https://www.fass.se/LIF/product?userType=2&nplId=20120726000024
64	Fluticasone propionate + Salmeterol	1 dose	2.75		https://www.fass.se/LIF/product?userType=2&nplId=20030404000096
65	Folic acid	100 mg	122.07		https://www.fass.se/LIF/product?userType=2&nplId=20190930000059
66	Formoterol	1 dose	2.94		https://www.fass.se/LIF/product?userType=2&nplId=20040607002841
67	Fusidic acid	1 dose	90.34		https://www.fass.se/LIF/product?userType=2&nplId=19871029000078
68	Gabapentin	100 mg	0.69		https://www.fass.se/LIF/product?userType=2&nplId=20040220000019
69	Glatiramer acetate	1 dose	304.75		https://www.fass.se/LIF/product?userType=2&nplId=20040312000057
70	Gum sterculia	1 dose	2.12		https://www.fass.se/LIF/product?userType=2&nplId=19790126000050
71	Hexamethylenetetramine	100 mg	0.26		https://www.fass.se/LIF/product?userType=2&nplId=19730525000053
72	Hydrochlorothiazide	100 mg	7.49		https://www.fass.se/LIF/product?userType=2&nplId=20090128000055
73	Hydrocortisone	100 mg	20.54		https://www.fass.se/LIF/product?userType=2&nplId=20040430000014
74	Hydrocortisone	1 dose	64.03		https://www.fass.se/LIF/product?userType=2&nplId=19550129000019
75	Hydroxizine	100 mg	4.02	*	https://www.fass.se/LIF/product?userType=2&nplId=19570405000021
76	Ibuprofen	100 mg	0.25		https://www.fass.se/LIF/product?userType=2&nplId=20060817000079
77	Isosorbide mononitrate	100 mg	5.57		https://www.fass.se/LIF/product?userType=2&nplId=20160402000016
78	Ketobemidone	100 mg	48.44		https://www.fass.se/LIF/product?userType=2&nplId=19930507000075
79	Ketoprofen	100 mg	1.23		https://www.fass.se/LIF/product?userType=2&nplId=19830311000073
80	Lamotrigine	100 mg	5.01	*	https://www.fass.se/LIF/product?userType=2&nplId=19940128000101
81	Lamotrigine	100 mg	2.13	*	https://www.fass.se/LIF/product?userType=2&nplId=20071020000084
82	Lansoprazole	100 mg	6.39		https://www.fass.se/LIF/product?userType=2&nplId=20050330000069

83	Levetiracetam	100 mg	1.41		https://www.fass.se/LIF/product?userType=2&nplId=20000929000031
84	Levothyroxine	1 dose	0.75		https://www.fass.se/LIF/product?userType=2&nplId=19541213000015
85	Lidocaine	1 dose	73.14		https://www.fass.se/LIF/product?userType=2&nplId=19491123000011
86	Liothyronine	1 dose	2.29		https://www.fass.se/LIF/product?userType=2&nplId=19571219000016
87	Loperamide	100 mg	53.42		https://www.fass.se/LIF/product?userType=2&nplId=19900504000187
88	Loratadine	100 mg	13.35		https://www.fass.se/LIF/product?userType=2&nplId=20040130000062
89	Losartan	100 mg	2.04		https://www.fass.se/LIF/product?userType=2&nplId=20090220000113
90	Lymecycline	100 mg	1.00		https://www.fass.se/LIF/product?userType=2&nplId=20151024000041
91	Medroxyprogesterone acetate	100 mg	20.22		https://www.fass.se/LIF/product?userType=2&nplId=19890630000031
92	Melatonin	100 mg	139.04		https://www.fass.se/LIF/product?userType=2&nplId=20201001000097
93	Metformin	100 mg	0.12		https://www.fass.se/LIF/product?userType=2&nplId=20081009000110
94	Methadone	100 mg	26.56		https://www.fass.se/LIF/product?userType=2&nplId=20170718000051
95	Methotrexate	1 dose	186.16		https://www.fass.se/LIF/product?userType=2&nplId=20070906000026
96	Methylphenidate	100 mg	18.70	*	https://www.fass.se/LIF/product?userType=2&nplId=20021101000335
97	Methylphenidate	100 mg	60.54	*	https://www.fass.se/LIF/product?userType=2&nplId=20060525000019
98	Metoprolol	100 mg	1.64		https://www.fass.se/LIF/product?userType=2&nplId=19750418000076
99	Metoprolol	100 mg	1.81		https://www.fass.se/LIF/product?userType=2&nplId=20080425000032
100	Metronidazole	100 mg	1.11		https://www.fass.se/LIF/product?userType=2&nplId=20180529000027
101	Metronidazole	1 dose	76.73		https://www.fass.se/LIF/product?userType=2&nplId=19871029000016
102	Mirtazapine	100 mg	6.50	*	https://www.fass.se/LIF/product?userType=2&nplId=20070810000020
103	Moclobemide	100 mg	2.35	*	https://www.fass.se/LIF/product?userType=2&nplId=19891208000118
104	Modafinil	100 mg	4.50		https://www.fass.se/LIF/product?userType=2&nplId=20140311000024
105	Mometasone	1 dose	0.64		https://www.fass.se/LIF/product?userType=2&nplId=20100708000020
106	Mometasone	1 dose	82.68		https://www.fass.se/LIF/product?userType=2&nplId=20101222000015
107	Mometasone	1 dose	2.04		https://www.fass.se/LIF/product?userType=2&nplId=20110721000020
108	Mometasone	1 dose	0.64		https://www.fass.se/LIF/product?userType=2&nplId=20140703000014
109	Montelukast	100 mg	8.95		https://www.fass.se/LIF/product?userType=2&nplId=20080704000043
110	Morphine	100 mg	16.30		https://www.fass.se/LIF/product?userType=2&nplId=20130713000050
111	Naltrexone	100 mg	15.99		https://www.fass.se/LIF/product?userType=2&nplId=20150611000044
112	Naproxen	100 mg	0.41		https://www.fass.se/LIF/product?userType=2&nplId=19871029000092
113	Nystatin	1 dose	2.82		https://www.fass.se/LIF/product?userType=2&nplId=20131102000040
114	Omeprazole	100 mg	19.55		https://www.fass.se/LIF/product?userType=2&nplId=20090716000023
115	Ondansetron	100 mg	40.74		https://www.fass.se/LIF/product?userType=2&nplId=20200701000178
116	Oxazepam	100 mg	5.47	*	https://www.fass.se/LIF/product?userType=2&nplId=19880219000026
117	Oxycodone	100 mg	15.06		https://www.fass.se/LIF/product?userType=2&nplId=20180716000038
118	Paracetamol	100 mg	0.07		https://www.fass.se/LIF/product?userType=2&nplId=20190301000022
119	Paracetamol + Codeine	1 dose	1.24		https://www.fass.se/LIF/product?userType=2&nplId=20090916000021
120	Paroxetine	100 mg	13.12	*	https://www.fass.se/LIF/product?userType=2&nplId=20080703000051
121	Phenoxymethylpenicillin	100 mg	0.40		https://www.fass.se/LIF/product?userType=2&nplId=19790831000024
122	Povidone	1 dose	58.81		https://www.fass.se/LIF/product?userType=2&nplId=19971219000151
123	Prednisolone	100 mg	27.30		https://www.fass.se/LIF/product?userType=2&nplId=19730831000037
124	Pregabalin	100 mg	9.03	*	https://www.fass.se/LIF/product?userType=2&nplId=20040607000731
125	Pregabalin	100 mg	4.10	*	https://www.fass.se/LIF/product?userType=2&nplId=20171110000076
126	Progesterone	100 mg	5.10		https://www.fass.se/LIF/product?userType=2&nplId=20181017000017
127	Promethazine	100 mg	6.50	*	https://www.fass.se/LIF/product?userType=2&nplId=19530228000028
128	Propiomazine	100 mg	5.18	*	https://www.fass.se/LIF/product?userType=2&nplId=19600628000017
129	Propranolol	100 mg	10.86		https://www.fass.se/LIF/product?userType=2&nplId=19651125000024
130	Propylene glycol	1 dose	240.66		https://www.fass.se/LIF/product?userType=2&nplId=20200512000022
131	Propylene glycol	1 dose	371.63		https://www.fass.se/LIF/product?userType=2&nplId=20030804004007
132	Pyridostigmine	100 mg	9.41		https://www.fass.se/LIF/product?userType=2&nplId=19550216000014
133	Quinagolide	1 dose	11.89		https://www.fass.se/LIF/product?userType=2&nplId=19931203000048
134	Ramipril	100 mg	26.17		https://www.fass.se/LIF/product?userType=2&nplId=20100615000052
135	Rituximab	1 dose	2,437.70		https://www.fass.se/LIF/product?userType=2&nplId=19980602000012
136	Salbutamol	1 dose	0.57		https://www.fass.se/LIF/product?userType=2&nplId=19970606000033
137	Salbutamol	1 dose	0.42		https://www.fass.se/LIF/product?userType=2&nplId=20061003000019
138	Salbutamol	1 dose	0.29		https://www.fass.se/LIF/product?userType=2&nplId=19980529000010
139	Sertraline	100 mg	1.19	*	https://www.fass.se/LIF/product?userType=2&nplId=20041110000249
140	Simvastatin	100 mg	2.70		https://www.fass.se/LIF/product?userType=2&nplId=20041018000303
141	Sodium chloride	100 mg	0.54		https://www.fass.se/LIF/product?userType=2&nplId=20210816000017
142	Sulfamethoxazole + Trimethoprim	1 dose	2.95		https://www.fass.se/LIF/product?userType=2&nplId=19770909000071
143	Sumatriptan	1 dose	334.79		https://www.fass.se/LIF/product?userType=2&nplId=19911018000020
144	Sumatriptan	100 mg	19.01		https://www.fass.se/LIF/product?userType=2&nplId=20070201000028
145	Sumatriptan	1 dose	59.60		https://www.fass.se/LIF/product?userType=2&nplId=19961108000058
146	Terbutaline	1 dose	0.85		https://www.fass.se/LIF/product?userType=2&nplId=19920214000024
147	Tiotropium bromide	1 dose	4.86		https://www.fass.se/LIF/product?userType=2&nplId=20060830000018
148	Tramadol	100 mg	1.94		https://www.fass.se/LIF/product?userType=2&nplId=20170201000049
149	Tranexamic acid	100 mg	0.54		https://www.fass.se/LIF/product?userType=2&nplId=19690328000028
150	Valaciclovir	100 mg	0.54		https://www.fass.se/LIF/product?userType=2&nplId=20070801000022
151	Venlafaxine	100 mg	1.18	*	https://www.fass.se/LIF/product?userType=2&nplId=20160401000024
152	Vortioxetine	100 mg	115.39	*	https://www.fass.se/LIF/product?userType=2&nplId=20120924000062
153	Zolmitriptan	100 mg	1,494.47		https://www.fass.se/LIF/product?userType=2&nplId=20011130000093
154	Zolpidem	100 mg	8.04	*	https://www.fass.se/LIF/product?userType=2&nplId=20011214000520
155	Zopiclone	100 mg	10.33	*	https://www.fass.se/LIF/product?userType=2&nplId=20081114000012

Notes: PPU = price per unit in SEK; Prices per pack were collected from Fass on December 18, 2023 and converted to either a price per 100 mg or a price per dose. R = * denotes that the product was indicated for PTSD, depression, anxiety, sleeping problems, bipolar disorder or ADHD.

Table S.2 Unit costs for healthcare utilisation

Type	TIC-P item(s)	Unit cost (SEK)
Visit/consultation with physician	1,(5),6	4,794
Homevisit from any healthcare staff	2	4,432
Visit/consultation with psychologist	(3),4	4,155
Visit/consultation with physiotherapist	7,(10)	2,330
Visit/consultation with counselor	8	3,249
Visit/consultation for addiction (DRG T61) with any healthcare staff	9	5,132
Visit with psychologist in outpatient day care	(11),12	2,932
Hospitalisation (per day)	13	19,632

Notes: Unit costs were collected for 2022 from the SALAR cost-per-patient data base, available at https://skr.se/skr/halsasjukvard/ekonomiavgifter/kostnadperpatientkpp/ (accessed 2024-01-05). In our analyses, the reported unit costs were inflated to the 2023 price level using Statistics Sweden's consumer price index for health care (1452.03/1390.13=1.0445). TIC-P items (see Table 1 for item numbers) within parentheses were not publicly funded; unit costs were applied to these items in a sensitivity analysis.
