# Supplementary material for: Cost-Effectiveness of App-Guided Self-Management for Posttraumatic Stress: Trial-Based Economic Evaluation
Source: J Med Internet Res. 2025 Sep 18;27:e69426. doi: 10.2196/69426 (PMC12491891; doi:10.2196/69426)
Supplement: Multimedia Appendix 2 [file jmir_v27i1e69426_app2.docx]

**Table S.3** Value of information in sensitivity analyses of the cost effectiveness of PTSD Coach versus no app-guided self-management ^a^

| Analysis | | EVPI | EVPPI_E_ | EVPPI_C_ |
| --- | --- | --- | --- | --- |
| Main analysis | | 5,417,642 | 12 | 4,937,613 |
| **Estimation** | |  |  |  |
|  | Diff-in-diff | 16,065,625 | 0 | 15,731,980 |
|  | Baseline adj | 3,896,541 | 0 | 3,726,946 |
|  | Pre-post | 142,384 | 0 | 118,950 |
| **Attrition**^b^ | |  |  |  |
|  | Complete cases | 14,880,790 | 308 | 14,087,485 |
|  | Negative attrition | 24,660,328 | 5,760,170 | 23,830,783 |
|  | Positive attrition | 4,037,788 | 757 | 3,595,780 |
| **Miscellaneous** | |  |  |  |
|  | Multiple imputation | 5,278,545 | 9 | 4,800,522 |
|  | Incl private care | 22,738,906 | 257,296 | 21,799,943 |
|  | More users | 19,663,629 | 45 | 17,921,337 |
|  | Higher app cost | 5,452,067 | 13 | 4,970,039 |

^a^ EVPI = expected value of perfect information; EVPPI = expected value of partial perfect information (on incremental effectiveness or incremental costs).

^b^ In the negative attrition scenario, the 95^th^ percentile of healthcare consumption and the 5^th^ percentile of HRQoL was used to impute missing observations for participants lost to follow up; the percentiles were reversed in the positive attrition scenario.
